# New Perspectives in the Adaptive Assessment of Depression: The ATS-PD Version of the QuEDS

**DOI:** 10.3389/fpsyg.2018.01101

**Published:** 2018-07-06

**Authors:** Andrea Spoto, Francesca Serra, Ivan Donadello, Umberto Granziol, Giulio Vidotto

**Affiliations:** ^1^Quantitative Psychology Laboratory, Department of General Psychology, University of Padua, Padua, Italy; ^2^Fondazione Bruno Kessler, Trento, Italy

**Keywords:** adaptive psychological assessment, formal psychological assessment, depression, qualitative and quantitative assessment, item response theory (IRT)

## Abstract

Measurement is a crucial issue in psychological assessment. In this paper a contribution to this task is provided by means of the implementation of an adaptive algorithm for the assessment of depression. More specifically, the Adaptive Testing System for Psychological Disorders (ATS-PD) version of the Qualitative-Quantitative Evaluation of Depressive Symptomatology questionnaire (QuEDS) is introduced. Such implementation refers to the theoretical background of Formal Psychological Assessment (FPA) with respect to both its deterministic and probabilistic issues. Three models (one for each sub-scale of the QuEDS) are fitted on a sample of 383 individuals. The obtained estimates are then used to calibrate the adaptive procedure whose performance is tested in terms of both efficiency and accuracy by means of a simulation study. Results indicate that the ATS-PD version of the QuEDS allows for both obtaining an accurate description of the patient in terms of symptomatology, and reducing the number of items asked by 40%. Further developments of the adaptive procedure are then discussed.

## 1. Introduction

Measurement in Psychology is a challenging issue that rose since the very beginning of the history of Psychology as a science. The first formalization of measurement in Psychology is due to the empirical research of Weber and to the Psychophysics of Fechner (1860), while Spearman (1904) paved the way for the measurement of theoretical constructs through methodologies such as factor analysis. The lack of a consistent formulation of the measurement problem in psychology was first addressed by Stevens by means of direct methods for psychological measurement (Stevens, 1946, 1951, 1957). An axiomatic definition of the measurement scales appeared within the theoretical framework of the Relational Theory of Measurement (RTM; Suppes and Zinnes, 1963; Suppes et al., 1989; Narens and Luce, 1993).

Currently, in psychological measurement the classical test theory (CTT; Spearman, 1904; Novick, 1965; Gulliksen, 2013) and the item response theory (IRT; Rasch, 1960; Lord, 1980) are the formal and methodological frameworks for the construction of measurement tools. The classical test theory relies on the evaluation of the reliability, validity and factorial structure of a defined psychological measure. An important limit lies in the impossibility to distinguish and compare the parameters related to the individuals (abilities) and those relative to the items (difficulties). On the other hand, the item response theory and the Rasch model (Rasch, 1960) explain the test performance of individuals by referring to the presence of latent traits. A relation between the latent traits and the observed scores is postulated, so that information on the first ones are inferred starting from the observable performance of the individual in answering the set of items. The relationship between test score and latent trait is expressed by a mathematical model, defined *a priori*. IRT models are a family of mathematical models which describe a wide number of contexts starting from the simple logistic model. The interest in applying these models is growing thanks to the numerous advantages compared to the classical test methods. IRT models represent a fundamental formal tool for applying adaptive measurement in psychology since they allow the definition of precedence relations among items according to their location on the latent trait dimension (Marsman et al., 2018).

In recent years another approach to measurement has been adopted in psychological assessment. This approach allows for expanding the measurement properties obtained through IRT by considering complex precedence relations among items (i.e., beyond the linear order). It refers to an axiomatic formulation of the relations among sets of items and sets of attributes investigated by them. It refers to the possibility of depicting the relations among items according to well established mathematical tools such as *lattices* and *posets* (Birkhoff, 1937, 1940; Davey and Priestley, 2002). It is the Formal Psychological Assessment (FPA; Spoto, 2011; Spoto et al., 2013a; Bottesi et al., 2015; Serra et al., 2015, 2017). It was developed with the aim of providing detailed information about the clinical features endorsed by a patient who answered a specific set of items of a questionnaire.

In the present article, this last methodology is employed to implement an adaptive algorithm for the assessment of depression. More specifically, the main aim of this article is to present the adaptive form of the Qualitative-Quantitative Evaluation of Depressive Symptomatology questionnaire (QuEDS; Serra et al., 2017).

The paper is structured as follows: In section 2, the general concepts concerning recent developments in psychological assessment are addressed; In section 3 a brief outline of the main deterministic and probabilistic issues related to the FPA is presented; section 4 introduces the main concepts of the adaptive algorithm implemented in this study; In sections 5 and 6 a simulation study aimed at testing the accuracy and the efficiency of the adaptive procedure is presented; Final remarks, limitations and future perspectives are explored in section 7.

## 2. Psychological assessment: state of the art

Measurement is a crucial issue in many fields of psychology. One of them is psychological assessment. The main tools adopted for carrying out measurement in psychological assessment are self-report questionnaires, observation and interviews. Clinical interviews and observation have the capacity for gathering and deepening several information such as nonverbal aspects, which are essential to make a diagnosis (Annen et al., 2012; Fiquer et al., 2013; Girard et al., 2014). This last is the main aim of clinical assessment, and therefore, the main goal of measurement in clinical psychology. For example, negative emotions and social behaviors are indicators of the severity of depression and relevant predictors of its clinical remission (Philippot et al., 2003; Uhlmann et al., 2012) that are beyond the control and awareness of the patient (Andersen, 1999; Geerts and Brüne, 2009). Nonetheless, both observation and clinical interview are time consuming and prone to inferential errors by clinicians (Strull et al., 1984; Nordgaard et al., 2013).

The self-report questionnaires provide scores that are supposed to indicate the severity of the symptomatology and the impairment level (Groth-Marnat, 2009). The score of a questionnaire is helpful in distinguishing individuals with critical clinical features, but it is not sufficient, in the form so far provided by both CTT and IRT, to differentiate patients with different symptom configurations who obtained similar scores (in the limit, the same score) to the test (Spoto et al., 2013a; Bottesi et al., 2015; Serra et al., 2015, 2017). Moreover, not all the items have the same “weight” from the clinical point of view, since they reflect different symptoms that may be more or less severe (Gibbons et al., 1985; Serra et al., 2017).

Therefore, measurement in clinical psychology should attempt to evaluate individual data in a broad perspective and it should account for individual specific features (Meyer et al., 2001). For example, the construct of depression, as represented by the score, can sometimes be misleading. Indeed, depression can manifest with a variety of different symptoms that may be due to a different culture or a different etiology (Benazzi et al., 2002; Goodwin and Jamison, 2007). Only through personalized assessment it is possible to clearly distinguish among such different manifestations of the disorder (Groth-Marnat, 2009; Serra et al., 2017). For these reasons, an increasing number of self-report assessment tools are validated according to the IRT framework (Gibbons et al., 2008; Embretson and Reise, 2013). In this way the assessment can be much more focused on the objective measure of the uniqueness of a particular clinical configuration. For instance, Balsamo et al. (2014) applied Rasch analysis to the item selection for the Teate Depression Inventory, a self-report depression tool; it has been highlighted by a number of papers that this tool, built according to the IRT methodology, performs better than tools developed within the CTT with respect to many different measurement properties such as, for instance, convergent-divergent validity (e.g., Innamorati et al., 2013; Balsamo et al., 2015a,b).

As mentioned above, IRT is also a crucial stepping stone for implementing adaptive testing, which in turn is an important way to implement the administration of a questionnaire in a personalized fashion. Each individual is administered with different scale items on the basis of the specific answers he/she provided to the previous ones (Wainer, 2000; Fliege et al., 2005). Within this field, Computerized Adaptive Testing (CAT; Wainer, 2000) is an approach which presents multiple advantages. Different studies showed that questionnaires could be shortened without loss of information by means of CAT, achieving a more efficient and equally accurate assessment (Petersen et al., 2006). CAT's procedure mimics the semi-structured interview (i.e., clinical interview where only some items, out a list, are posed according to specific adaptive selection criteria), letting the algorithm to carry out inferences by accounting for all the information collected and following a logically correct process (Spoto, 2011). It should be quite easy to understand why the assessment of knowledge is one of the core areas in which such procedures have been developed. For instance, Eggen and Straetmans (2000) combined IRT with statistical procedures, like sequential probability ratio test and weighted maximum likelihood, for classifying people under exam. Other systems use Bayesian statistical techniques instead of IRT in the evaluation of students' knowledge. Examples are EDUFORM (Nokelainen et al., 2001), and PARES (Marinagi et al., 2007). In the field of knowledge assessment the ALEKS (Assessment and LEarning in Knowledge Spaces) system implements the theoretical framework of Knowledge Space Theory (KST; Doignon and Falmagne, 1985, 1999; Falmagne and Doignon, 2011) for the adaptive assessment of the so called *knowledge state* of a student, i.e., the set of items that he/she is able to solve about a specific topic (Grayce, 2013).

The formulation of an adaptive algorithm is clearly more difficult in the clinical setting. In fact, the objectivity of the questions and therefore of the answers given by the subject is much more questionable, and the probability of misinterpretations in the answers is increased. Despite this, research has demonstrated that both IRT and CAT (Baek, 1997) can be applied to the measurement of attitudes and personality variables (Reise and Waller, 1990). In the clinical framework, Spiegel and Nenh (2004) developed an expert system, which calculates possible symptom combinations given the answers of a patient and returns all possible diagnoses coherent with such combination. Yong et al. (2007) developed an interactive self-help system for depression diagnosis that provides advice about patients levels of impairment. Simms et al. (2011) developed the CAT for Personality Disorders (CAT-PD) aimed at realizing a computerized adaptive assessment system. CAT has been applied also in developing adaptive classification tests by means of stochastic curtailment using CES-D for depression (Finkelman et al., 2012; Smits et al., 2016). Gibbons et al. (2008) used the combination of item response theory and CAT in mood and anxiety disorder assessment. In particular, they applied a bi-factor structure consisting of a primary dimension and four sub-factors (mood, panic-agoraphobia, obsessive-compulsive, and social phobia) to build the CAT version of the Mood and Anxiety Spectrum Scales (MASS; Dell'Osso et al., 2002). Results of this study showed that the adaptive tool allowed to both administer a small set of the items (the most relevant for a given individual) with no loss of information compared to the classical form of the MASS, and strongly reduce time consumption as well as patient and clinician burden. In six patients with mood disorders (three major depressive disorder and three bipolar disorder) who were interviewed by the psychiatrist, many of the CAT items investigating important information, such as a history of manic symptoms, potentially risky behaviors, etc., were both endorsed and not documented in the psychiatric evaluation through SCID-I (First et al., 1996). Gibbons' study is an important example of how adaptive testing can be effective and efficient.

Although there have been several attempts to apply adaptive clinical assessment, as far as we know, no system is able to combine adaptivity, quantitative and qualitative information, and punctual estimates of error parameters. Within clinical psychology, the Formal Psychological Assessment (Spoto et al., 2010, 2013a; Serra et al., 2015; Granziol et al., 2018) represents an important contribution in the improvement of adaptive psychological assessment, allowing to overcome the obstacles encountered up to now in this field. The main deterministic and probabilistic concepts of this methodology are presented in the next section.

## 3. The formal psychological assessment

An adaptive testing, most of the time, relies on a formal substratum composed by several relations among items (Donadello et al., 2017). The FPA is a methodology which allows defining assessment tools able to detect specific symptoms in several mental disorders, independently of the kind of assessment used, such as self-report (Serra et al., 2015, 2017) or behavioral observations (Granziol et al., 2018). FPA makes it possible applying two theories of Mathematical Psychology in psychological assessment: The Knowledge Space Theory (KST; Doignon and Falmagne, 1999; Falmagne and Doignon, 2011) and the Formal Concept Analysis (FCA; Wille, 1982; Ganter and Wille, 1999). The core characteristic of FPA is the definition of a relation between a set of items and a set of clinical criteria. In the next two subsections are reported separately the deterministic and probabilistic main concepts of FPA.

### 3.1. FPA deterministic concepts

In FPA methodology, a very basic concept is the *clinical domain*, intended as a nonempty set *Q* of questions that can be asked to a patient for investigating a certain psychopathology. Each item is referred as an *object*. The complete list of the items included in the QuEDS, namely the objects in the present article, are listed in Table 1.

**Table 1:** The list of items of the QuEDS grouped by sub-scale.

**Item**	**Description**
	**Cognitive scale**
QuEDS5	I am stressed by feelings of guilt
QuEDS6	I think the world is cruel and unhappy
QuEDS9	I think my life is hell and I only deserve to feel bad
QuEDS10	I feel incapable of facing life's events
QuEDS14	I feel incapable and totally useless
QuEDS19	I have thought about killing myself
QuEDS20	Sometimes I think it would be better if I were dead
QuEDS21	I am really worried about my health
QuEDS24	I am afraid about everything that will happen to me because I am incapable
QuEDS25	I feel like I don't have any more power over my empty and sad life
QuEDS27	Making choices is hard for me
QuEDS30	I feel I am a burden other people and it would be better if I killed myself
QuEDS32	I am disappointed in myself and the choices I have made
QuEDS33	I have problems making decisions
QuEDS41	I often feel like a loser
	**Somatic scale**
QuEDS1	I feel like I don't have the same energy to have sex
QuEDS2	I often wake up in the middle of the night and I can't go back to sleep
QuEDS3	I feel like that my thinking is slowing down
QuEDS4	I have sleep problems
QuEDS11	I suffer from somatic disorders (e.g., headache, stomach ache)
QuEDS13	I am less interested in sex
QuEDS16	My desire to eat is not the same
QuEDS22	My weight has had significant changes
QuEDS23	I've visibly lost (or gained) weight
QuEDS26	My appetite has changed
QuEDS28	I feel I'm slowing down in my daily routines
QuEDS31	I don't have much energy and I feel tired
QuEDS35	My ability to think and memorize has been reduced
QuEDS39	I feel so tired and without any energy that I need help to look after myself
	**Affective scale**
QuEDS7	I keep crying very easily
QuEDS8	I get irritated very easily
QuEDS12	I have lost interest in the future which doesn't have anything good for me
QuEDS15	I see the same unhappiness I have now in the future
QuEDS17	I often feel like crying, but I cannot do it
QuEDS18	I can't have interest and pleasure in people and things as before
QuEDS29	I feel helpless and inhibited facing my incapacity to concentrate
QuEDS34	I feel sad
QuEDS36	I don't have any interest and desire to do anything
QuEDS37	I am agitated about the idea that this sadness won't ever leave me
QuEDS38	I feel agitated
QuEDS40	I am better in the evening more than in the morning

For instance, the item QuEDS34 “I feel sad” is an object. For the sake of simplicity, the term item will be preferred to object in the sequel. The subset *K*⊆*Q* of all the items that are endorsed by a patient is called the *clinical state* of that patient. Each item investigates one or more *attributes*, intended as a diagnostic criteria of a psychopathology selected from either clinical sources like the DSM-5 (American Psychiatric Association, 2013) or the scientific literature, or both. The complete list of the attributes investigated by the QuEDS is displayed in Table 2.

**Table 2:** The list of the attributes investigated by the items of the QuEDS.

**Attribute**	**Description**
A1	Depressed mood
A2	Diminished interest and pleasure
A3	Decreased interest in sex
A4	Increase or loss of weight
A5	Gain or loss of appetite
A6	Insomnia or hypersomnia
A7	Agitation
A8	Psycho-motor retardation
A9	Fatigue or energy loss
A10	Feelings of worthlessness (or Beck's negative view of self)
A11	Feelings of guilt
A12	Diminished ability to think and concentrate
A13	Indecision
A14	Recurrent thoughts of death
A15	Suicidal ideation or attempted suicide
A16	Beck's negative view of the world
A17	Beck's negative expectation of the future
A18	Seligman's learned helplessness
A19	Irritability
A20	Apathy
A21	Health concern
A22	Somatic disorders
A23	More positive mood in the evening

For instance, the diagnostic criterion “Depressed mood” of the DSM-5 is investigated by the aforementioned item. The collections of both items and attributes make it possible to build the so called *clinical context*, formally a triple (*Q, M, I*), where *Q* is the set of items, *M* is a set of attributes and *I* is a binary relation between the sets *Q* and *M* which assigns to each item *q* ∈ *Q* the attributes *m* ∈ *M* it investigates. The clinical context can be represented as a Boolean Matrix, having the items in the rows and the attributes in the columns: whenever an item *q* investigates an attribute *m* (i.e., whenever the relation *qIm* holds true), the *qm* cell contains the value 1, otherwise 0.

Starting from the clinical context, the *clinical structure* K can be delineated (Spoto et al., 2010, 2016). A clinical structure is a collection of clinical states, containing at least the empty set (∅) and the whole clinical domain (*Q*) and it represents the implications among the items of the domain. Whenever a clinical structure is closed under set union (i.e., $K_{1}\cup K_{2}\in K$ for all $K_{1},K_{2}\in K$) it is a *clinical space*. On the other hand, a clinical structure closed under intersection (i.e., $K_{1}\cap K_{2}\in K$ for all $K_{1},K_{2}\in K$), it is a *clinical closure space*. In order to obtain a structure where all the states *K* are in a one to one connection with the set of attributes endorsed by all the items in *K*, it is necessary to modify the clinical context by defining a relation *R* between items and attributes, which is dual to *I*:

$$\begin{array}{l} qRm\Leftrightarrow q\neg Im. \end{array}$$

According to this relation, a clinical closure space is obtained, where each state is in a one to one correspondence with the set of attributes endorsed by the items in each state (for details refer to Spoto et al., 2010). In other words the relation *R* allows for representing in the structure a principle often used in clinical and medical practices: if a patient endorses an item, he should present all the attributes investigated by that item. From a practical point of view, a clinical structure is useful since it includes only the clinical states, that is all and only the admissible response patterns given the clinical context. Any state K∈K is coherent with the theoretical framework and, therefore, it does not violate specific order relation among items. In FPA, the relation required is based on the attributes investigated by each item and it is called *prerequisite relation*, stating that whenever an item *q* investigates a subset of attributes of another item *r*, *q* is a prerequisite for *r*. For instance, taken the subset of QuEDS's selected attributes composed by {A1, A17} and two items of the QuEDS, namely QuEDS34 which investigates only A1, and QuEDS15, investigating both A1 and A17. The two rows of the clinical context representing QuEDS15 and QuEDS34 will be in an inclusion relation with respect to the attributes investigated (Table 3).

**Table 3:** The precedence relation between items QuEDS15 and QuEDS34 as depicted in the clinical context.

**Items**	**A1**	**…**	**A17**	**…**	**A23**
QuEDS15	1	…	1	…	0
QuEDS34	1	…	0	…	0

Among the following response patterns:

- {∅};
- {*QuEDS*34};
- {*QuEDS*15};
- {*QuEDS*34, *QuEDS*15},

only the patterns a, b, and d are clinical states. In fact, it is not possible (excluding errors) that a patient who endorses item QuEDS15 does not endorse item QuEDS34 (i.e., the pattern c). A clinical structure can be represented as a complete lattice displaying the partial order among the items of a domain, where each node contains a subset of items (investigating a specific subset of attributes; Granziol et al., 2018). In the case at hand, the clinical structure is a clinical closure space where each node contains the items endorsed by the patient and the uniquely determined set of attributes (symptoms) corresponding to that set of questions.

By delineating a clinical structure, it is possible to obtain the deterministic skeleton for defining a computerized adaptive assessment, which needs to be completed also from a probabilistic point of view. The next section will deepen the probabilistic features needed to implement the algorithm.

### 3.2. FPA probabilistic concepts

A deterministic clinical structure provides a fundamental starting point for the procedure aimed at creating an adaptive assessment. Nonetheless, such a structure is incomplete from both a theoretical and practical point of view. In fact, each state could be present with different frequencies in the population; moreover, the observed response pattern of a subject could not represent his/her real state. The probability of observing each clinical state π_*K*_ is then related to both its actual frequency in the population, and to two further parameters, namely the *false negative* (β) and the *false positive* (η). The false negative refers to the probability that the patient does not endorse an item that he/she actually presents. The false positive parameter, on the contrary, refers to the probability that a patient endorses an item that he/she does not present. By means of all the aforementioned parameters (i.e., π_*K*_ for each K∈K; β_*q*_ and η_*q*_ for each item *q* ∈ *Q*) a *probabilistic clinical structure* (Donadello et al., 2017) can be obtained. Formally, it is a triple (Q,K,π) where (Q,K) is the clinical structure and π is the probability distribution on K estimated through a sample of patients (Spoto et al., 2010). The probability distribution for each response pattern *R*⊆*Q* is obtained by means of a response function assigning to *R* its conditional probability given a state *K* (for all states K∈K), as displayed by the unrestricted latent class model represented by Equation 1:

(1) $$\begin{array}{l} P\left(R\right)=\underset{K\in K}{\sum }P\left(R|K\right)\pi \left(K\right). \end{array}$$

This model is the so called *basic local independence model* (BLIM; Falmagne and Doignon, 1988; Doignon and Falmagne, 1999). Within the probabilistic clinical structure, the responses to the items are assumed to be locally independent. The conditional probability *P*(*R*|*K*) is determined by the probability of false negative (β_*q*_) and false positive (η_*q*_) while answering to *q*, as displayed by Equation (2):

(2) $$\begin{array}{l} P\left(R|K\right)=\left(\underset{q\in K\backslash R}{\prod }\beta_{q}\right)\left(\underset{q\in K\cap R}{\prod }\left(1-\beta_{q}\right)\right)\left(\underset{q\in R\backslash K}{\prod }\eta_{q}\right)\left(\underset{q\in \bar{R\cup K}}{\prod }\left(1-\eta_{q}\right)\right). \end{array}$$

In the present study, the expectation-maximization algorithm (Dempster et al., 1977) has been used in order to estimate both the β and η parameters and the probability distribution for K. These estimates have been carried out on a sample of 383 individuals, according to the same procedure employed in, e.g., Spoto et al. (2013a), Bottesi et al. (2015), and Donadello et al. (2017). Such estimates were then used to implement the adaptive algorithm whose general functioning is detailed in the next section.

## 4. The adaptive testing system for psychological disorders algorithm

In this section we aim at introducing the Adaptive Testing System for Psychological Disorders Donadello et al. (ATS-PD; 2017) developed starting from the clinical structure and the parameters' estimate via the BLIM.

Within this framework, the clinical structure is the deterministic skeleton defining the starting point for an adaptive assessment which, if no error is assumed, could reasonably proceed as follows:

- Select the item that is closest to be present in 50% of the clinical states of the structure;
- Ask this item to the patient;
- Register the dichotomous answer;
- Exclude all the states not containing the investigated item if the answer is “yes,” or vice versa, all the states that contain the item if the answer is “no.”

These steps are repeated on the remaining states until only one state remains. The output is the clinical state with all the attributes (diagnostic criteria) satisfied by all the items of the state. This procedure applied in a real context would almost surely fail due to the absence of a probabilistic model defining both the probabilities of the different states, and the error probabilities for the answers. As it has been shown in the previous section, the probabilistic model which accounts for both these issues could be the BLIM. Therefore, an adaptive procedure should make an appropriate use of the probabilities of the states (π_*K*_) and of the false positive (η) and false negative (β) rates for each item.

Thus, the above outlined procedure can be therefore modified as follows:

- Detect and administer the item *q* which best splits into two equal parts the probability mass of the states (*questioning rule*), and register the dichotomous answer;
- Update the probability π_*K*_ of all the states K∈K according to the following *updating rule*:
  - if an affirmative answer to *q* is observed: increase π_*K*_ for all K∈K which contain *q*, and decrease π_*K*_ for the remaining states;
  - if a negative answer to *q* is observed: decrease π_*K*_ for all K∈K which contain *q*, and increase π_*K*_ for the remaining states.
- Repeat the previous steps until a given condition is satisfied (*stopping rule*).

The algorithm used in this research implements these three main steps. The questioning rule selects the item to ask, i.e., the item *q* ∈ *Q* that is “maximally informative.” This characteristic is satisfied by the item(s) for which the sum of π_*K*_ for all the states containing *q* best approaches 0.50. In other words, this item maximizes the obtainable information irrespectively of its observed answer (i.e., either “yes” or “no”). If many items are equally informative, one of them is chosen at random. We call *L*_*n*_(*K*) the probability of the state *K* at the step *n*. At each step of the procedure, the subject's response is collected by the system. Then, the updating rule is applied to obtain the likelihood *L*_*n*+1_(*K*) for all the states K∈K. More precisely, let us denote an affirmative response with *r* = 1 and a negative one with *r* = 0. It is then possible to formalize the updating rule of the probability *L*(*K*) for each K∈K as follows:

(3) $$\begin{array}{l} L_{n+1}\left(K\right)=\frac{\zeta^{K}L_{n}\left(K\right)}{\underset{K^{\prime }\in K}{\sum }\zeta^{K}L_{n}\left(K^{\prime }\right)} \end{array}$$

where

(4) $$\begin{array}{l} \zeta_{q,r}^{K}=\left\{ \begin{array}{cc} \zeta_{q,1}\; & \text{if}q\in K\text{,}r=1;\\ 1\; & \text{if}q\notin K\text{,}r=1;\\ 1\; & \text{if}q\in K\text{,}r=0;\\ \zeta_{q,0}\; & \text{if}q\notin K\text{,}r=0.\\ \; \end{array}\right. \end{array}$$

In this formulation ζ is a parameter always greater than 1 that increases the likelihood and influences the efficiency of the adaptive assessment process. The higher ζ, the more reliable are considered the answers provided by the subject, and therefore, the more efficient (but potentially less accurate) the adaptive procedure. It has been observed by Falmagne and Doignon (2011) that ζ values less than 2 make the assessment redundant. On the contrary, fixing the ζ value to an excessively high number could affect algorithm accuracy. It has been proven that an adequate value of ζ could be 21 (Falmagne and Doignon, 2011). This value allows for an accurate and efficient detection of the state of individuals in several applications, e.g., ALEKS (Falmagne et al., 2013). An alternative way to estimate ζ is based on the η_*q*_ and β_*q*_ parameters of each item (see Falmagne and Doignon, 2011, p. 265). The estimate is carried out according to the following formulas:

$$\begin{array}{ll} \zeta_{q,1}=\frac{1-\beta_{q}}{\eta_{q}} & \zeta_{q,0}=\frac{1-\eta_{q}}{\beta_{q}} \end{array}$$

This rule is local since it takes into account both the η and β of the last item asked in order to update the probability of the states. According to this method the “weight” of each item in updating the probabilities is a function of its error rates. Namely, an item whose error rates are low (i.e., whose answer is more reliable) will produce a significant modification on the probability distribution of the states, while a less reliable item will have a weaker effect in updating of the probabilities of the states.

In order to further refine the updating of the states probabilities given the pattern observed at a specific step *n* of the adaptive assessment, a Bayesian rule can be introduced according to what described by Donadello et al. (2017):

(5) $$\begin{array}{l} P\left(K_{i}|R\right)=\frac{P\left(R|K_{i}\right)L_{n}\left(K_{i}\right)}{\sum_{j\;=\;1}^{\left|K\right|}P\left(R|K_{j}\right)L_{n}\left(K_{j}\right)} \end{array}$$

Where *P*(*R*|*K*_*i*_) is obtained by Equation (2), and *L*_*n*_(*K*) is the estimated probability of the state *K* at the step *n*.

All these steps are replicated until a given stopping criterion is reached. In the present article, the stopping rule is satisfied whenever *L*_*n*_(*K*_*q*_) is outside the interval [0.20, 0.80] for all *q* ∈ *Q*. In this way the algorithm stops as soon as any possible item to be asked splits the probability mass in very unequal parts, indicating that it is almost surely either inside or outside the state. This choice is coherent with previous literature (Falmagne and Doignon, 2011, p. 362). When the stopping criterion is matched, the algorithm concludes the assessment and provides as output the response pattern *R*, the estimated state *K* (with its estimated probability) and the amount of time needed to complete the assessment.

In the next section, we will present a simulation study aimed at testing the algorithm under different conditions in order to identify the best performing configuration of the procedure. Before conducting such a simulation we estimated the parameters of the BLIM in order to provide the deterministic skeleton with probabilistic weights.

## 5. A simulation study

Testing the adaptive procedure with respect to both its accuracy and efficiency is a necessary operation in order to guarantee that (i) the information collected through the adaptive form of the questionnaire is reliable, and (ii) that the administration time of the questionnaire is actually reduced. In order to reach these goals, the traditional form of the QuEDS containing 41 dichotomous items (“Yes”/“No”) grouped into three sub-scales (namely: Cognitive, Somatic and Affective) was administered to a sample of 383 individuals. Using the collected data, the parameters of the BLIM were estimated. Then, such values were passed to the adaptive procedure. Finally, the adaptive algorithm, under different conditions, simulated the administration of the test starting from the available 383 response patterns, and the results were analyzed. The details of all these passages are provided below.

### 5.1. Sample

The sample of 383 Italian individuals included a clinical group consisting of 38 subjects with Major Depressive Episode (who were diagnosed with either major depressive disorder or bipolar disorder). These patients were recruited by the Neurosciences Mental Health and Sensory Organ (NESMOS) Department of La Sapienza University, Rome. The psychiatrists of NESMOS Department evaluated the presence of Major Depressive Episode in participants of the clinical group by means of the clinical interview and the SCID-I. The diagnosis was then formulated according to the DSM-IV-TR nosology classification system. The exclusion criteria were mental retardation and psychotic traits in order to guarantee a correct interpretation of the meaning of QuEDS items. The 47% of participants were males and the remaining 53% were females. The majority of the participants had a high school diploma, and their age ranged between 21 and 69 years old (*M* = 33.5; *SD* = 4.8). The remaining 345 individuals were randomly selected from the general population and recruited in Padova (68% were females). The majority of participants had a high school diploma, and their age ranged between 19 and 58 years old (*M* = 27.5; *SD* = 6.4). Participants of the non-clinical group did not undergo a psychological assessment. Before the beginning of the administration of the test they were asked to indicate whether they were currently under pharmacological or psychotherapeutic treatment for MDE. The exclusion criterion in the non-clinical group was the presence of MDE (i.e., individuals under pharmacological or psychotherapeutic treatment for depression).

The study was conducted in accordance with the Declaration of Helsinki and the research protocol was approved by the Psychology Ethical Committee of the University of Padua. All participants entered the study of their own free will and provided their written informed consent before taking part. They were informed in detail about the aims of the study, the voluntary nature of their participation, and their right to withdraw from the study at any time and without being penalized in any way. Furthermore, participants were allowed for asking the restitution about their own score, providing authors with their own auto generated code, used during the administration phase.

### 5.2. Procedure

All participants completed informed consent and sociodemographic forms before answering the questionnaire items. All participants completed the written form of QuEDS according to the following instructions: “Please answer “Yes” or “No” to the following statements on the basis of how you felt in the last 15 days.” No time limit was imposed. Clinical participants provided written, informed consent for potential research analysis and anonymous reporting of clinical findings in aggregate form, at clinical intake.

### 5.3. Parameters estimate

As mentioned in the previous sections, the estimate of BLIM's parameters (i.e., π_*K*_ for each state, β_*q*_ and η_*q*_ for each item), as well as of the fit of the model, were performed with a specific version of the Expectation-Maximization Algorithm (Dempster et al., 1977). For the details of the algorithm, refer to Spoto (2011). The tested models were obtained from the formal contexts displayed in Tables 4–6 according to the methodology described in Spoto et al. (2010).

**Table 4:** The clinical context for the Cognitive sub-scale of the QuEDS.

**Items**	**A1**	**A10**	**A11**	**A12**	**A13**	**A14**	**A15**	**A16**	**A17**	**A18**	**A21**
QuEDS5	0	0	1	0	0	0	0	0	0	0	0
QuEDS6	0	0	0	0	0	0	0	1	0	0	0
QuEDS9	1	1	1	0	0	0	0	1	0	0	0
QuEDS10	0	0	0	0	0	0	0	0	0	1	0
QuEDS14	0	1	1	0	0	0	0	0	0	1	0
QuEDS19	0	0	0	0	0	1	1	0	0	0	0
QuEDS20	0	0	0	0	0	1	0	0	0	0	0
QuEDS21	0	0	0	0	0	0	0	0	0	0	1
QuEDS24	0	0	0	0	0	0	0	0	1	1	0
QuEDS25	1	0	0	0	0	0	0	1	0	1	0
QuEDS27	0	0	0	1	1	0	0	0	0	0	0
QuEDS30	1	1	1	0	0	1	0	1	0	0	0
QuEDS32	0	1	1	0	0	0	0	0	0	0	0
QuEDS33	0	0	0	0	1	0	0	0	0	0	0
QuEDS41	0	1	0	0	0	0	0	0	0	0	0

**Table 5:** The clinical context for the Somatic sub-scale of the QuEDS.

**Items**	**A3**	**A4**	**A5**	**A6**	**A7**	**A8**	**A9**	**A12**	**A18**	**A20**	**A22**
QuEDS1	1	0	0	0	0	0	1	0	0	0	0
QuEDS2	0	0	0	1	1	0	0	0	0	0	0
QuEDS3	0	0	0	0	0	1	0	1	0	0	0
QuEDS4	0	0	0	1	0	0	0	0	0	0	0
QuEDS11	0	0	0	0	0	0	0	0	0	0	1
QuEDS13	1	0	0	0	0	0	0	0	0	1	0
QuEDS16	0	0	1	0	0	0	0	0	0	0	0
QuEDS22	0	1	0	0	0	0	0	0	0	0	0
QuEDS23	0	1	0	0	0	0	0	0	0	0	0
QuEDS26	0	0	1	0	0	0	0	0	0	0	0
QuEDS28	0	0	0	0	0	1	1	0	0	0	0
QuEDS31	0	0	0	0	0	0	1	0	0	0	0
QuEDS35	0	0	0	0	0	1	1	1	0	0	0
QuEDS39	0	0	0	0	0	1	1	0	1	1	0

**Table 6:** The clinical context for the Affective sub-scale of the QuEDS.

**Items**	**A1**	**A2**	**A7**	**A12**	**A17**	**A19**	**A20**	**A23**
QuEDS7	1	0	0	0	0	1	0	0
QuEDS8	0	0	0	0	0	1	0	0
QuEDS12	0	1	0	0	1	0	0	0
QuEDS15	1	0	0	0	1	0	0	0
QuEDS17	1	0	0	0	0	0	1	0
QuEDS18	0	1	0	0	0	0	1	0
QuEDS29	0	0	0	1	0	0	1	0
QuEDS34	1	0	0	0	0	0	0	0
QuEDS36	0	1	0	0	0	0	1	0
QuEDS37	1	0	1	0	0	0	0	0
QuEDS38	0	0	1	0	0	0	0	0
QuEDS40	0	0	0	0	0	0	0	1

The fit of each of the three models has been tested by Pearson's Chi-square. It is well established that for large data matrices (as those used in the present study) the asymptotic distribution of χ^2^ is not reliable. Therefore, a *p*-value for the obtained χ^2^ was calculated by parametric bootstrap with 5,000 replications. An important fit index is provided by the estimates of the error rates. In general, they are expected to be low, but it is crucial that for each item the following inequality holds: η_*q*_ < 1−β_*q*_. If this condition is not satisfied, then the assessment loses its meaning, since the probability of observing a false positive (η) on an item *q* would be greater than the probability of observing an actual affirmative answer to *q*. Spoto et al. (2012) established a specific connection between the characteristics of the context and the identifiability of the error parameters of the items. In this respect the value of the unidentifiable parameters (Spoto et al., 2013b; Stefanutti et al., 2018) was fixed to a constant corresponding to the maximum possible value of the parameter (Stefanutti et al., 2018) in order to both preserve the computability of the adaptive procedure and adopt the maximally conservative approach from a diagnostic point of view, preferring accuracy to efficiency.

This first step of the study provided the needed parameters for calibrating the adaptive procedure.

### 5.4. Simulation design

Six different conditions for testing the adaptive algorithm were generated by manipulating the following two variables:

1. Estimate of ζ parameter (2 levels): (i) 21 in one case; (ii) estimated via the values of η and β in the second;
2. Implementation of Bayesian updating rule (3 levels): (i) on-line (at each step *n* of the adaptive procedure), (ii) off-line (when the stopping criterion is reached), (iii) absent (no Bayesian updating is applied).

The adaptive procedure was then run according to each of the six conditions described above in order to simulate the 383 response patterns collected in the previous part of the study. The task of the algorithm was to administer the QuEDS in an adaptive form and provide the clinical state as output.

In order to check for the accuracy and the efficiency of the procedure, a number of indexes were used. First, the average number of items asked to converge (i.e., to match the stopping criterion) was used to test the efficiency of the ATS-PD version of QuEDS in terms of reduction of the number of items administered to a patient. Second, the distance between the reconstructed state and the paper and pencil pattern observed for each specific patient was used to evaluate procedure's accuracy. In fact, the higher the distance between the reconstructed state and the response pattern, the greater is the amount of information that is inconsistent between the two modalities of administration of the test. This, in turn, may be due to the error parameters of the items, to a misspecification of the model, or to problems with the algorithm. Since the first two options are excluded given the good fit and the acceptable error estimates (described in the previous section), a strong divergence between the observed pattern and the reconstructed state could be due to some errors in the algorithm; therefore, the measured distance is expected to be as low as possible if the algorithm is accurate. In this respect some further concepts need to be introduced.

A response pattern *R*_*i*_ is the list of the observed answers provided by a subject *i* to the written version of QuEDS. *K*_*i*_ is the state in K that is the output of the adaptive assessment when the input is the response pattern *R*_*i*_. It is important to emphasize that the adaptive procedure always produces a state K∈K as output even if the observed response pattern $R_{i}\notin K$. Thus, we define the distance *d*(*K*_*i*_, *R*_*i*_) as the cardinality of *K*_*i*_Δ*R*_*i*_. As a consequence, the results of the simulation for each subject can fall into one of the following mutually exclusive categories:

1. *K*_*i*_ = *R*_*i*_: this happens when the response pattern $R_{i}\in K$. In this case *d*(*K*_*i*_, *R*_*i*_) = 0. In the specific condition, the output of the adaptive assessment is exactly the same of the output obtained with the written version of the sub-scale;
2. *K*_*i*_ ≠ *R*_*i*_: This happens whenever $R_{i}\notin K$. In this case of course, *d*(*K*_*i*_, *R*_*i*_) > 0. In this situation two alternatives may occur:
  - *d*(*K*_*i*_, *R*_*i*_) is minimum, that is, there is no $K^{*}\in K$ such that $d\left(K^{*},R_{i}\right)<d\left(K_{i},R_{i}\right)$;
  - *d*(*K*_*i*_, *R*_*i*_) is not minimum, that is, there exists $K^{*}\in K$ such that $d\left(K^{*},R_{i}\right)<d\left(K_{i},R_{i}\right)$.

Of course, the occurrence of this last situation should be as rare as possible if the adaptive algorithm is accurate.

## 6. Results

Results are presented separately for the model fitting analysis, and for the algorithm efficacy and effectiveness test.

### 6.1. Model fitting

It is important to stress that the size of the three structures was relatively small counting 124 states for the Cognitive scale, 163 for the Somatic scale, and 142 for the Affective scale. The results of the model fitting for the three structures demonstrated an adequate fit of the models to the set of collected data [Cognitive: $\chi_{\left(32,144\right)}^{2}=23,348$, bootstrap-*p* = 0.07; Somatic: $\chi_{\left(15,972\right)}^{2}=7,237$, bootstrap-*p* = 0.16; Affective: $\chi_{\left(3,928\right)}^{2}=8,696$, bootstrap-*p* = 0.06]. Therefore, a general adequate fit of the structures was observed.

Another fundamental information obtained by the model fitting was the estimate of both the η and β parameters for each item of the scale. With respect to the η parameters, the estimated values are in general adequately small for almost all items, ranging between 0.01 and 0.18. Only few items presented relatively high values of the estimated β. For the Cognitive scale such items are QuEDS9 with β = 0.50, and QuEDS30 with β = 0.44. This criticism may be explained by the phrasing of the items which both include two coordinate sentences. In the Affective scale of QuEDS two items reported high β estimates: namely item QuEDS7 with β = 0.44, and item QuEDS17 with β = 0.45. Interestingly both these items are related to crying, suggesting that either the subjects could intentionally fake the specific answer, or that subjects' answer could be affected by a poor introspection about “crying.” Although not fully satisfying, it will be shown in the next section that these values did not affect the performance of the adaptive procedure.

Given the number of participants and the number of states, the estimate of the π_*K*_ for some states K∈K was 0. Therefore, we did not use such information in the following part of the simulation and we fixed the starting values of all π_*K*_ in the adaptive procedure according to the uniform distribution. This specific implementation is quite common in several applications.

In general, both the fit indexes and the parameters' estimates were satisfactory and were, then, implemented in the adaptive procedure whose performance is analyzed in the next subsection.

### 6.2. Clinical state reconstruction

The results of both accuracy and effectiveness tests supported the goodness of the adaptive algorithm. First of all (and actually, as expected) the system, in all the tested versions, was able to correctly reproduce the patient's pattern whenever the pattern *R*_*i*_ was a state in K. Moreover, for the great majority of the patterns, whenever $R_{i}\notin K$, the algorithm mapped the pattern into the closest state in the structure, thus, *d*(*K*_*i*_, *R*_*i*_) = *min* in most cases. Only in a limited number of cases happened that the algorithm mapped a response pattern $R_{i}\notin K$ to a state that was not at the minimum distance, thus, for some cases happened that there was a state $K_{i}^{*}\in K$ such that $d\left(K_{i}^{*},R_{i}\right)<d\left(K_{i},R_{i}\right)$. This could depend on the sequence of questions asked by the system, which in turn is affected by the error parameters, and by the type of update used by the system. However, this situation has rarely occurred in the simulations.

Table 7 summarizes the results with respect to both accuracy and efficiency of the algorithm.

**Table 7:** Main results of the adaptive algorithm testing.

		**Cognitive**		**Somatic**		**Affective**	
**ζ**	**Bayesian update**	**Max items**	**Not Min**	**Max items**	**Not Min**	**Max items**	**Not Min**
21	off-line	17	12	9	8	8	40
21	on-line	11	15	9	6	8	35
21	absent	17	11	9	8	8	39
(η_*q*_, β_*q*_)	off-line	20	7	11	8	23	39
(η_*q*_, β_*q*_)	on-line	**11**	**10**	**9**	**5**	**8**	**29**
(η_*q*_, β_*q*_)	absent	20	7	11	8	29	34

The first column contains the levels of the variable ζ; The second column refers to the implementation of the Bayesian update. The remaining columns contain, for each sub-scale, the maximum number of questions asked to reach the stopping criterion, and the number of response patterns mapped to a state K_i_ such that there exists $d\left(K^{*},R_{i}\right)<d\left(K_{i},R_{i}\right)$ for the three sub-scales. In bold the best performing configuration.

The table displays that the best performing configuration of the ATS-PD version of the QuEDS is the one with on-line Bayesian update and the parameter ζ computed as a function of η_*q*_ and β_*q*_. In the cognitive scale, which has 15 items in total, with this configuration we had a maximum of 11 questions asked and a minimum of 7 to reach the stopping criterion; the average is 8.83 items asked (*SD* = 0.47). It means that the saving in terms of question posed is between 31 and 53%. We found 10 response patterns *R* in which the distance *d*(*K*_*i*_, *R*_*i*_) was not minimal. In the specific case, $d\left(K_{i},R_{i}\right)-d\left(K_{i}^{*},R_{i}\right)\leq 2$. It means that the distance between the output state *K*_*i*_ and the state $K_{i}^{*}$ that was the closest one to *R*_*i*_ was never greater than 2 (that is, no more than two answers in *K*_*i*_ were different from $K_{i}^{*}$, whose distance was minimum from *R*_*i*_).

The somatic scale has 14 items in total. In the best performing configuration we observed a maximum of 9 items asked to reach the stopping criterion and a minimum of 8 item asked to achieve the output of the assessment; the average is 8.42 items asked (*SD* = 0.82). The saving in terms of questions posed is between 36 and 50%. Out of the 173 observed different response patterns, five were mapped to a state whose distance from the pattern was not minimal. Also in this case $d\left(K_{i},R_{i}\right)-d\left(K_{i}^{*},R_{i}\right)\leq 2$.

The affective scale counted a total 12 items in the written version. In the best performing configuration we had a maximum of 8 items asked and a minimum of 7; the average is 7.66 items asked (*SD* = 0.47). The saving in terms of questions posed is between 33 and 42%. In this scale 29 response patterns were mapped to a state at a non minimal distance. In the specific case $d\left(K_{i},R_{i}\right)-d\left(K_{i}^{*},R_{i}\right)\leq 3$. This last scale seemed to perform in a less accurate, although still adequate and effective, way.

It is important to stress how the procedure is carried out on-line: this means that the questioning rule, the updating rule (together with the Bayesian correction) and the stopping rule are applied in real time even on a standard machine. This indicates an adequate optimization of the computational costs of the procedure.

## 7. Discussion

This paper aimed at presenting the adaptive version of the three sub-scales (namely, cognitive, somatic, affective) of the QuEDS questionnaire. The computerized algorithm was implemented for the new questionnaire based on an extension of an already existing algorithm for the assessment of knowledge (Doignon and Falmagne, 1999; Falmagne and Doignon, 2011). The parameters of the probabilistic model (i.e., π_*K*_ for each clinical state K∈K, η_*q*_ and β_*q*_ for every *q* ∈ *Q*) were estimated through an iterative procedure based on maximum likelihood (Dempster et al., 1977) on data from the 383 participants. The estimated parameters were then used to calibrate the adaptive algorithm. The simulation study was carried out to test the efficiency and accuracy of the implemented adaptive procedure. Results supported that the adaptive version of the QuEDS provides clinicians with accurate information collected in an efficient way. Moreover, the information collected by means of the adaptive version of QuEDS allows the differentiation of individuals with the same score but with different symptoms (i.e., with different clinical states) and, possibly, different severity of the episode. These properties represent a relevant improvement in the amount, and quality of the collected diagnostic information, as well as in the amount of time needed for case formulation.

The parameter estimates provided the starting point for the implementation of the questioning rule and of the updating rule. This last was tested under different conditions with respect to the computation of the multiplicative parameter ζ. Finally, the opportunity to apply a Bayesian update was tested. Results showed that the most efficient and accurate implementation of the algorithm included the estimate of ζ via the η and β parameters, and the application of an on-line Bayesian updating.

It is important to highlight how the adaptive version of the questionnaire allows for a consistent reduction of the number of questions asked. In the classical written form of the QuEDS, each participant had to answer all 41 items, 15 for the cognitive sub-scale, 14 for the somatic sub-scale, 12 for the affective sub-scale. In the adaptive form of QuEDS only a percentage ranging between 50 and 70% of the items is asked.

The present study and the adaptive version of the QuEDS presents some limitations which should be addressed in future research. Although the sample size is adequate to obtain reliable estimates of the error parameters of the items and of the model fit, it is too small to achieve reliable estimates of the clinical states' probabilities. In fact, given the size of the three structures (respectively 124, 163, and 142 states) and the obtained error parameters of the items, a reliable estimate of the π_*K*_ parameters would need a sample of approximately 1,000 individuals. Notice that this limitation is not crucial since, in general, with large structures the *a priori* probability of each state is very low, thus in the adaptive form, the possibility of starting from a uniform distribution on the states is not that strong and generally accepted. The second limitation of the present study is the relatively low number of patients in the clinical sub-sample. This limitation, which could appear critical in the perspective of classical methodologies in psychological measurement, within the framework of FPA is not that crucial, since the estimate of the parameters and the fit involve the sample in the whole rather than taking into account different sub-samples. Nonetheless, the recruitment of a greater number of patients to refine the estimates and the efficiency-efficacy of the adaptive version of the QuEDS will be the subject matter of future research. One final limitation deserves mention: The present version of the QuEDS does not contain any control scale for social desirability. The inclusion of social desirability scale into self-report tools for the assessment of depression is an important and debated issue (e.g., Langevin and Stancer, 1979; Pichot, 1986; Tanaka-Matsumi and Kameoka, 1986; Cappeliez, 1990; Balsamo and Saggino, 2007) and it will receive further attention in future research in order to provide users of the QuEDS with complete information about this specific issue.

To conclude, this new form of the QuEDS allows a clinician to differentiate the individual's depressive symptoms beyond the score and to administer only the items related to its symptomatology following the logical flow of question-answer. Thus, two patients who obtain the same score to the test can be treated differently according to their symptoms, since answering the same number of items does not mean having the same symptoms configuration.

The future directions of the development of the adaptive version of the QuEDS questionnaire are twofold: on the one hand it is necessary to improve the user interface in order to achieve a simple graphical output able to provide the clinician with a helpful and accessible way to interact with the system. On the other hand, the formal definition of the suggestions for further investigation on the patient have to be formalized and implemented. This last issue is in continuity with the operational approach adopted by the Cognitive Behavioral Assessment 2.0 (CBA 2.0; Bertolotti et al., 1990; Sanavio et al., 2008), and represents the fundamental philosophical approach implemented by FPA methodology. Furthermore, several refinements of ATS-PD system can be implemented, for example the possibility of simplifying the updating rule for real-time application of QuEDS as implemented by Augustin et al. (2013). Another important future direction will be the extension of this approach to the case of polytomous items. The implementation of these extensions would allow FPA to be used with Likert scales, promoting its wider application in both psychological measurement and clinical practice.

## Author contributions

AS defined the framework of the paper, conducted the parameter estimates analysis and prepared the final version of the manuscript. FS created the items of the questionnaire, supervised the definition of the formal contexts and contributed to the introduction and discussion of the paper. ID implemented the adaptive algorithm and tested the accuracy and efficiency of the procedure. UG prepared the description of the main deterministic and probabilistic issues of Formal Psychological Assessment, and prepared part of the introduction. GV supervised the whole research project.

### Conflict of interest statement

The authors declare that the research was conducted in the absence of any commercial or financial relationships that could be construed as a potential conflict of interest.
